# Light-RCV: a lightweight read coverage viewer for next generation sequencing data

**DOI:** 10.1186/1471-2105-16-S18-S11

**Published:** 2015-12-09

**Authors:** Che-Wei Chang, Wen-Bin Lee, An Chen-Deng, Tsunglin Liu, Joseph T Tseng, Darby Tien-Hao Chang

**Affiliations:** 1 Department of Electrical Engineering, National Cheng Kung University, Tainan 70101, Taiwan; 2 Institute of Bioinformatics and Biosignal Transduction, National Cheng Kung University, Tainan 70101, Taiwan

## Abstract

**Background:**

Next-generation sequencing (NGS) technologies has brought an unprecedented amount of genomic data for analysis. Unlike array-based profiling technologies, NGS can reveal the expression profile across a transcript at the base level. Such a base-level read coverage provides further insights for alternative mRNA splicing, single-nucleotide polymorphism (SNP), novel transcript discovery, etc. However, to our best knowledge, none of existing NGS viewers can timely visualize genome-wide base-level read coverages in an interactive environment.

**Results:**

This study proposes an efficient visualization pipeline and implements a lightweight read coverage viewer, Light-RCV, with the proposed pipeline. Light-RCV consists of four featured designs on the path from raw NGS data to the final visualized read coverage: i) read coverage construction algorithm, ii) multi-resolution profiles, iii) two-stage architecture and iv) storage format. With these designs, Light-RCV achieves a < 0.5s response time on any scale of genomic ranges, including whole chromosomes. Finally, a case study was performed to demonstrate the importance of visualizing base-level read coverage and the value of Light-RCV.

**Conclusions:**

Compared with multi-functional genome viewers such as Artemis, Savant, Tablet and Integrative Genomics Viewer (IGV), Light-RCV is designed only for visualization. Therefore, it does not provide advanced analyses. However, its backend technology provides an efficient kernel of base-level visualization that can be easily embedded to other viewers. This viewer is the first to provide timely visualization of genome-wide read coverage at the base level in an interactive environment. The software is available for free at http://lightrcv.ee.ncku.edu.tw.

## Background

Current next-generation sequencing (NGS) technologies have provided biologists with an unprecedented scale of genomic data that require analysis [1,2]. Instead of reporting a single expression value for each transcript in array-based profiling technologies, NGS technologies can reveal the read count variation within a transcript at the base level. Such a base-level read coverage provides further insights for analyzing alternative mRNA splicing, single-nucleotide polymorphism (SNP), novel transcript discovery, etc [3,4].

Constructing a base-level read coverage requires alignment of numerous reads on the reference genome. Read alignments are difficult to interpret by human. Many NGS viewers, such as Artemis [5], Savant [6], Tablet [7] and Integrative Genomics Viewer (IGV) [8], have been developed to visualize read alignments into friendly graphic profiles. Some of these NGS viewers can depict a base-level read coverage but only in a small scale; while some of them provide a genome-wide read coverage but not at the base level. To our best knowledge, none of existing NGS viewers can timely visualize a genome-wide base-level read coverage in an interactive environment. The considerable data scale and computational complexity pose a challenge to develop such tools.

To address this challenge, this study proposes an efficient visualization pipeline for NGS data and implements a lightweight read coverage viewer, Light-RCV, with the proposed pipeline. The pipeline consists of four featured designs on the path from read alignments to the final visualized read coverage. The four designs are critical to immediate visualization (i.e. the response time is shorter than 0.5 second) of a base-level read coverage. Light-RCV was implemented as an offline program with web technology. Most researchers prefer not to upload their NGS data to a remote server. An offline program fulfills this requirement. On the other hand, web technology was chosen because it is suitable for embedding in other web-based NGS tools and is familiar to most biologists. Other offline NGS tools also can embed Light-RCV on top of a native browser component, which is supported by major programming languages such as the WebBrowser class in C#, C++, F# and VB, the WebView class in Java (for Android devices), the WebView class (for OSX devices) and the UIWebView class (for iOS devices) in Objective-C.

## Results and discussion

This section introduces the interface of Light-RCV and reports the results of a performance evaluation. Finally, the results of a case study are presented.

### User interface

**Figure 1:**
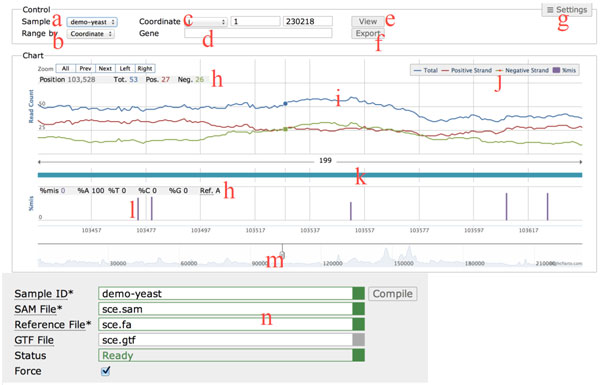
**Appearance of Light-RCV**. The user interface of Light-RCV: (a) ***Sample***: switching among different compiled NGS data / (b) ***Range by***: an option for specify a genomic range by coordinate range or by a gene name / (c) ***Coordinate***: chromosome number/name, start position and end position / (d) ***Gene***: a search box for gene name / (e) ***View***: button to show the read coverage in the specified range / (f) ***Export***: saving the current view to an image file / (g) ***Setting***: used to show/hide the compilation panel / (h) Detailed information of the point hovered by the mouse / (i) Main plot of the read coverages / (j) Legend of the plot for showing/hiding the tracks / (k) Annotation track, would show if gtf file is provided at the compilation stage / (l) Bar chart for the mismatch rate / (m) Navigator bar for zooming and scrolling the viewing range / (n) Compilation panel for compiling NGS data.

Figure 1 shows the appearance of Light-RCV. The main interface provides only a few controls for specifying a genomic range, which are the most frequently used operations. In internal usability tests, almost every first-time user could use Light-RCV to visualize NGS data without any instructions. The controls for the compilation stage, which is hidden in the main interface by default, are described in the end of this subsection.

To see the read coverage of a specific genomic range, the first step is to choose a compiled NGS data with the *Sample *control (Figure 1(a)). The package of Light-RCV attaches a compiled sample, demo-yeast, for users who have no NGS data at hand to experience Light-RCV. The second step is to specify a genomic range either by a coordinate range (the *Coordinate *control, Figure 1(c) or by a gene name (the *Gene *control, Figure 1(d)). This alternative is decided by the *Range by *control (Figure 1(b)). In practice, users need not to actually change the *Range by *control, which changes accordingly whenever users change the *Coordinate *or *Gene *control. Light-RCV provides many facilities to make controls behave naturally. For example, the coordinate start and end are automatically switched when the start is larger than the end. Genes can be specified by a gene symbol, name and alias. While typing, users can see the full gene names that fit the current input and select the desired one, namely "auto completion." After the genomic range is selected, clicking the *View *button (Figure 1(e)) brings the read coverage (Figure 1(i)) in that range. This can also be done by pressing the *Enter *key in keyboard. Clicking the *Export *button (Figure 1(f)) saves the current view to an image file.

Light-RCV shows three read coverages: *Total *for reads aligned to both positive and negative strands in each position; *Positive Strand *for reads aligned to the positive strand; *Negative Strand *for reads aligned to the negative strand. Below the three read coverages is a bar chart for the mismatch rate (*%mis*) of each position (Figure 1(l)), which is useful for detecting SNPs. The four tracks of information (*Total, Positive Strand, Negative Strand *and *%mis*) can be shown/hidden by the legends (Figure 1(j)). Below the four tracks is an annotation track (Figure 1(k)). When mouse hovers over a position, more detailed information are shown (Figure 1(h)). Note that the composition information is shown when the viewing range is smaller than about 500 bps (depending on the window size). Zooming in can be done by simply dragging in the chart or by the navigation bar (Figure 1(m)). The latter provides intuitive navigational operations (zooming in/out, scrolling, etc).

Finally, users can click the *Settings *button (Figure 1(g)) to show the controls for the compilation stage (Figure 1(n)). To compile an NGS experiment, one has to specify four data: i) *Sample ID *for identification, which would be shown in the *Sample *control (Figure 1(a)); ii) *SAM File*, which contains the alignments of NGS reads on a reference genome; iii) *Reference File*, which is a FASTA file containing the sequence of the reference genome; iv) *GTF File*, which contains gene coordinates and annotations. The *GTF File *is optional but is required for many controls such as Figure 1(d) and (k). In Light-RCV, specifying a GTF file is recommended. After specifying the data, clicking the *Compile *button starts the compilation stage. The status is shown in the *Status *control and the sample ID is shown in the *Sample *control after the compilation succeeds.

### Performance evaluation

This subsection compares the response time of Light-RCV and three popular offline NGS viewers. Table 1 shows the results, where values in parentheses indicate that the corresponding NGS viewer did not display a base-level read coverage. Savant and IGV do not display read coverages for genomic regions larger than 20 kilo base pairs (kb) and 70 kb, respectively. Tablet shows only summarized read coverages in which the read counts of 500 genomic positions are averaged to a value. These settings/limitations were designed for short response time and good user experience (UX). Light-RCV, on the other hand, aimed to achieve a shorter response time without these limitations.

**Table 1:** Time comparison of NGS viewers.

Frequency	Savant	Tablet	IGV	Light-RCV
Per NGS experiment^1^				
*Saccharomyces cerevisiae*	0.00 ± 0.00	0.00 ± 0.00	0.00 ± 0.00	56.12 ± 1.07
Per loading an NGS experiment^2^				
*Saccharomyces cerevisiae*	3.71 ± 0.22	11.79 ± 0.48	9.94 ± 0.55	0.00 ± 0.00
Per visualization of a genomic region^3^				
1 kb genomic region	0.44 ± 0.10	(1.08 ± 0.12)	1.37 ± 0.28	0.33 ± 0.02
2 kb genomic region	0.39 ± 0.09	(1.08 ± 0.16)	1.17 ± 0.13	0.33 ± 0.03
5 kb genomic region	0.53 ± 0.09	(1.01 ± 0.17)	1.10 ± 0.12	0.37 ± 0.01
10 kb genomic region	0.77 ± 0.08	(1.07 ± 0.13)	1.16 ± 0.11	0.38 ± 0.04
20 kb genomic region	0.90 ± 0.08	(1.03 ± 0.12)	1.14 ± 0.14	0.43 ± 0.03
50 kb genomic region	*	(1.02 ± 0.15)	1.63 ± 0.16	0.76 ± 0.03
.1 Mb genomic region	*	(0.84 ± 0.10)	*	1.34 ± 0.06
.2 Mb genomic region	*	(0.96 ± 0.12)	*	2.47 ± 0.12
.5 Mb genomic region	*	(0.92 ± 0.12)	*	12.37 ± 0.23
Amortized processing time				
20 kb genomic region	0.91 ± 0.08	(0.92 ± 0.12)	1.16 ± 0.14	0.53 ± 0.03

^1^Time required by the compilation stage of Light-RCV. Other NGS viewers did not have this stage. ^2^Time required when the user chooses an NGS data in viewers. ^3^Time required when the user specifies a genomic region, which is critical to user experience. *The viewer does not show read coverage at these region.

Time is measured in seconds. Values in parentheses indicate that the corresponding NGS viewer did not display a base-level read coverage. Tablet shows a summarized read coverage in which the read counts of 500 genomic positions are averaged to a value.

The first two sections in Table 1 ("Per NGS experiment" and "Per loading an NGS experiment") stand for the time required to prepare an NGS data. The preparation time of Light-RCV was longer than those of other NGS viewers, which is reasonable because Light-RCV moves as many computations as possible to this stage. Notably, the preparation of Light-RCV is conducted only once for an NGS experiment, while other NGS viewers have a startup delay of three to ten seconds whenever users load an NGS experiment. In addition to the startup time, the UX of an NGS viewer relies more on the response time of each genomic range change, which corresponds to "Per visualization of a genomic region" in Table 1. The response time of Light-RCV was less than half second [9] regardless of the genomic range. Strictly speaking, the read coverage in a large genomic range was not at the base level because of the limitation of screen resolution. Light-RCV smartly detected the screen width and returned only necessary data points. In this regard, screen width is a factor of the response time of Light-RCV. The numbers in Table 1 were measured in a 1920x1080 screen, which is a rather big screen in contemporary personal computers. The UX studies have shown that the response is considered immediate when the delay is shorter than half second. Namely, users feel immediate response after specifying a genomic region in Light-RCV. This immediate response time is shorter than those of IGV and Tablet in a genomic region smaller than a kilo base pairs (kb) and that of Savant on a genomic region smaller than 5 kb.

The efficiency of the entire process of converting the raw data to the final visualized read coverage can be estimated by amortizing the preparation time to each genomic position (the "Amortized processing time" in Table 1). The amortized time of a 20 kb region in Light-RCV was 0.53s (56.12s÷12.1 Mb×20 kb+0.43s), which is faster than the compared NGS viewers. This explains that the long preparation time of Light-RCV was due to computation arrangement but not performance deficiency. Table 2 shows that Light-RCV consumed the same scale of memory of other NGS viewers, which reveals that the speed of Light-RCV did not require the cost of a large cache. The efficient read coverage construction algorithm is the key to the amortized time. Furthermore, the two-stage architecture and the design of the internal format (which moved most computations to the first stage) enabled an immediate response time.

**Table 2:** Memory comparison of NGS viewers.

Size^1^	Savant	Tablet	IGV	Light-RCV
1 kb	309.6	(305.7)		44.6
2 kb	301.8	(310.5)		62.3
5 kb	294.9	(310.5)		67.8
10 kb	288.1	(312.5)		71.6
20 kb	282.2	(311.5)	70.0	80.8
50 kb	*	(312.5)	150.0	118.5
.1 Mb	*	(317.4)	*	193.9
.2 Mb	*	(317.4)	*	266.6
.5 Mb	*	(320.3)	*	389.6

The memory unit is megabyte (MB). ^1^Size of the genomic region to be visualized. Values in parentheses indicate that the corresponding NGS viewer did not display a base-level read coverage, where Tablet shows summarized profiles in which the read counts of 500 genomic positions are averaged to a value. *The viewer does not show read coverage at these regions.

Table 3 shows the distinctive features of Light-RCV in comparison with other NGS viewers. This table, which focuses on Light-RCV's features, demonstrates the uniqueness of Light-RCV but does not prove that Light-RCV is superior over other NGS viewers. Light-RCV lacks some features, such as array data support (expression, copy number, etc.), of other NGS viewers. Table 3 highlights that the largest contribution of Light-RCV is in processing read coverage. Another distinctive feature of Light-RCV is embeddable, which is a benefit of using web technology. To sum up, Light-RCV is light and fast. It focuses on the most important duty of a viewer: visualization. However, this does not indicate that Light-RCV is better or faster than other multi-functional NGS viewers, which may spend time for more analyses than visualization. Light-RCV should be considered a tool that complements other NGS viewers. Researchers can use other NGS viewers to analyze and use Light-RCV to see the data as shown in the following subsection.

**Table 3:** Feature comparison of NGS viewers.

	Savant	Tablet	IGV	Light-RCV
Read coverage				
Base level^1^	Yes	No	Yes	Yes
Whole chromosome^2^	Partial	Yes	Partial	Yes
Multi-resolution model^3^	Yes	No	Yes	Yes
Embeddable in other program^4^	No	No	No	Yes

^1^One point of Tablet read coverage curve represents 500 bps. ^2^Savant and IGV do not show read coverage when the viewing range is greater than 20 kb and 70 kb, respectively. ^3^Savant and IGV claimed to have multi-resolution model for all data types according to their documents. However, they did not clearly indicated that read coverage is included. Multi-resolution model is not visible to users. Instead, this technique is used to expedite visualization at all scales of viewing ranges. The time comparison under different viewing ranges in Table 1 provides a performance evaluation of the multi-resolution model. This model is a key to achieve both base-level read and whole chromosome read coverage in Light-RCV. However, the reasons why Savant and IGV limited the viewing range of read coverage are unknown. ^4^Embeddable is also not visible to regular users but is useful for developers.

### Case study

This subsection demonstrates a practical usage flow to show the importance of visualizing read coverage. This case was provided by our collaborative research group, which has used Light-RCV for several months to analyze NGS data.

**Figure 2:**
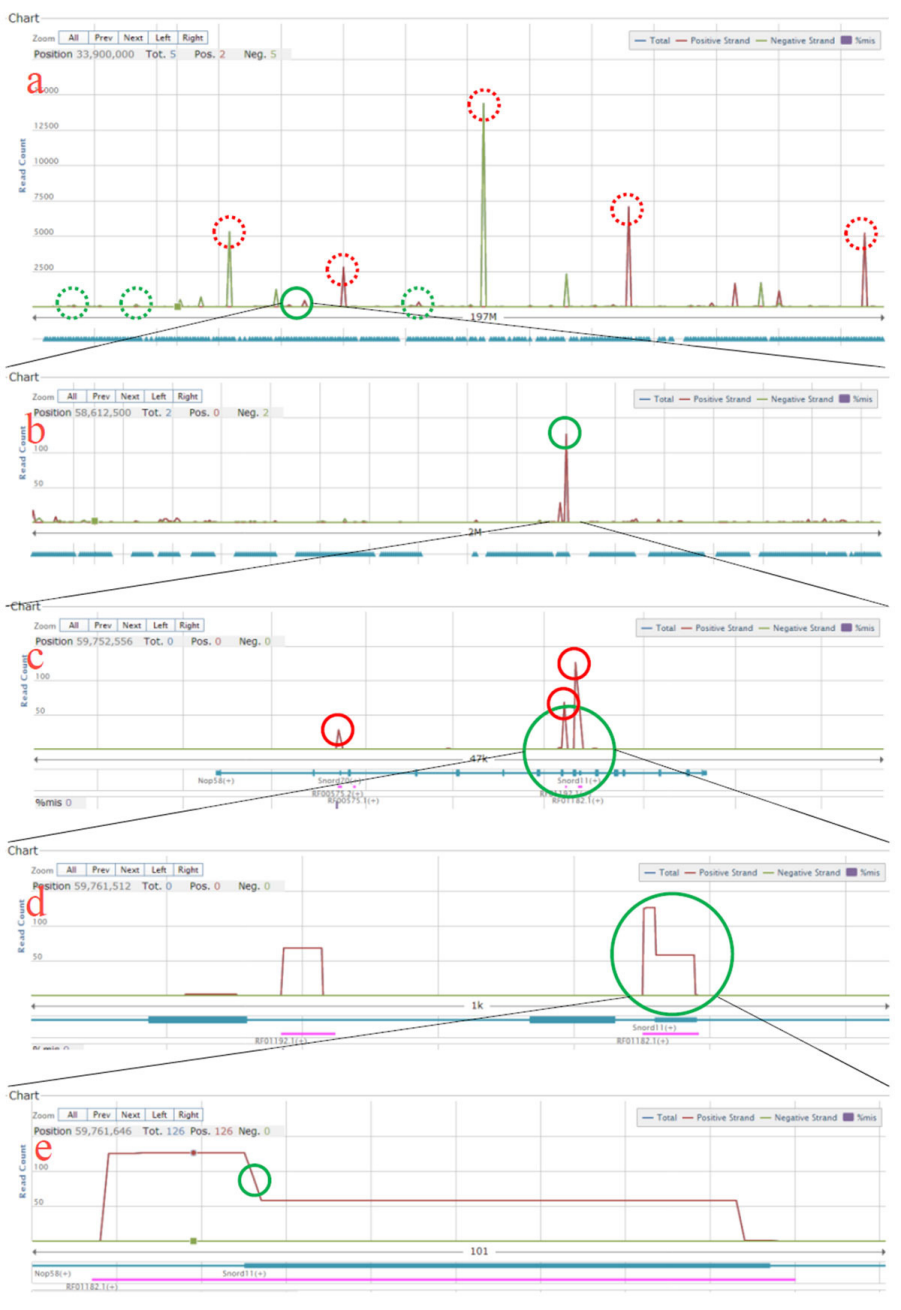
**Case study**. (a) to (e) show the zooming process discussed in the case study section.

The operator began the workflow from a read coverage at the whole chromosome level (Figure 2a, mouse chrI, about 197M). At this level, one might be attracted by the most sharp peaks (the red circles in Figure 2a). However, these peaks are easily identifiable by almost all analysis tools. In practical analyses, on the other hand, the operator was interested in less obvious peaks (green circles in Figure 2a) and analyzed them individually. In this case study, the area of the solid green circle was chosen.

After zooming into the ~2M area (Figure 2b), the operator identified a peak with a read count higher than 100 (the green circle in Figure 2b). The operator further zoomed into the peak. In this ~47k area (Figure 2c), the transcript annotations were shown. The operator got three clear read coverage peaks (the red circles in Figure 2c) and had some transcript candidates (Nop58, Snord70, RF00575.2,...) according to the annotation track below the read coverage. Cuffdiff (a program in the Cufflinks package)[10], one of the most widely used software for calculating gene expression from NGS data, incorrectly assigned these reads to gene Nop58 since the read coverage peaks were consistent with some exons (the thick lines) of Nop58. With the aid of the visualized read coverage, the operator quickly determined that the read count of Nop58 was a false positive. Many NGS viewers provide automatic analysis. However, for cases that need visualized read coverage, short response time is more important than comprehensive analyses.

The operator then zoomed into the right two peaks (the green circle in Figure 2c) and obtained a ~1k area, Figure 2d. At this level, the operator can see the shape of the read coverage. The irregular shape of the right transcript (the green circle in Figure 2d) attracted the operator.

Finally, the journey ended at a 101 bps area (Figure 2e), which reveals two facts. First, the boundaries of the read coverage peak were several bases smaller than those of the transcript RF01182.1. This reveals that the quality of the read alignments (performed by TopHat [11] in this case study) was relatively low at the ends of the transcripts. Second, there is a shorter transcript, Snord11, that overlaps with RF01182.1. The read coverage curve in Figure 2e has a clear decrease near the green circle, which is perfectly matched with the boundary of Snord11. This reveals the difficulty of automatically assigning read counts in areas with overlapped transcript annotations. Manual determination with the aid of a visualized read coverage, is a compromise solution for this problem at present.

In this case study, transcript RF01182.1 and Snord11 are basically the same transcript after the operator queried other databases such as Ensembl [12]. Therefore, this can be easily solved by the operator or, in another words, these is no need to solve. However, if the overlapped transcripts are different, the operator must conduct further analyses. The further analyses are various (case-by-case) and beyond the scope of this study.

In summary, Light-RCV provides a convenient tool for warning operators about these issues. The above workflow heavily relies on manual efforts. Most members of our collaborative research group agreed that the immediate response time of Light-RCV was critical for everyday analyses.

## Conclusions

This study proposed four designs on visualizing read counts of each genomic position. This efficient visualization pipeline was implemented as a lightweight read coverage viewer, Light-RCV, which aims at timely visualizing genome-wide base-level read coverages in an interactive environment. It achieved immediate response time and outstanding amortized time.

## Methods

The methods section is organized as follows. First, the web technologies used in Light-RCV are described. The second to fifth subsections describe Light-RCV's four distinctive designs in comparison with existing offline NGS viewers.

### Web technology

The web technologies used in Light-RCV can be divided as backend and frontend. The backend technologies handle data access/storage, which was developed with PHP and run on an Apache web server. The frontend technologies handle data visualization and user input, which was developed with HTML5, CSS3 and JavaScript and running on browsers. Because of the value of NGS data to individual researchers, Light-RCV was developed as an offline tool that can be run locally on a personal computer without network connection. Light-RCV is compatible with any portable web servers such as USBWebServer (http://www.usbwebserver.net/en/) and XAMPP (https://www.apachefriends.org/index.html). Users are given guidelines for quickly setting up a local web environment and do not have to upload their NGS data to a remote server.

### Read coverage construction algorithm

**Figure 3:**
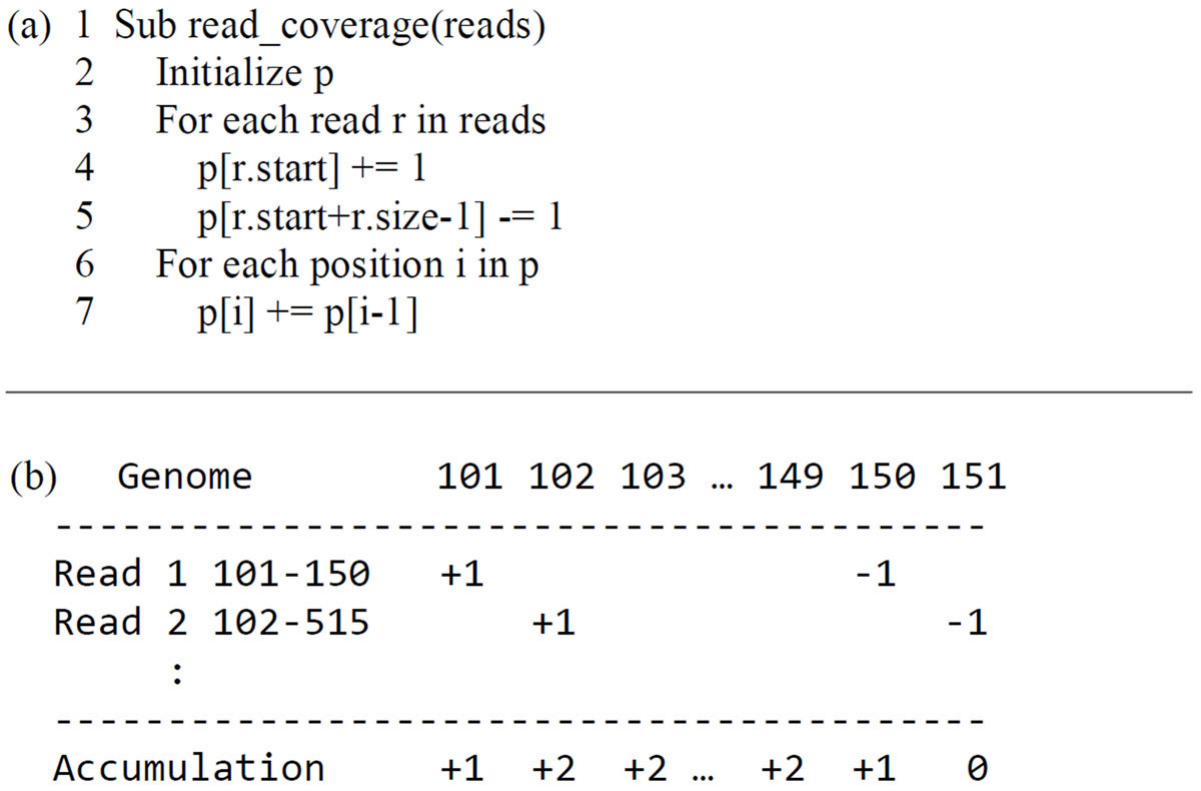
**Construction of base-level read coverage**. The algorithm for calculating base-level read coverages, demonstrated in (a) Pseudo code and (b) schematic plot. Notice that the for-loop of line 6 of (a) skips the first position.

The input of NGS viewers is a huge set of read alignments, which are usually stored in SAM or BAM files. Such files are not optimized for NGS viewers. The NGS viewers must convert the raw format into an internal format before read coverage visualization. Light-RCV has four featured designs on the path from read alignments to the visualized read coverage. The first one is the read coverage construction algorithm. The first step of converting read alignments (i.e., the start and end positions of reads on the genome) to the read counts of each position is to allocate a big array of the genome size. To process a read at position *i *of length *l*, a for-loop is then used to increase the elements *i, i*+1 ... *i*+l-*1 *of the big array by one. Light-RCV expedited the time complexity of processing a read alignment from *O*(*l*) to *O*(1) (Figure 3). In Light-RCV, only element *i *increases by one and element *i*+*l*-1 decreases by one when processing a read at position *i *of length l. Namely, the big array of the proposed read coverage construction algorithm stores the changes of the base-level read coverage before line 6 of Figure 3a. Lines 6 and 7 accumulate the changes in the base-level read coverage. For processing *r *reads on a genome with *g *base pairs, the time complexities of original for-loop and Light-RCV's method are *O(r*×*l) *and *O(r+g)*, respectively. The additional *O*(*g*) of Light-RCV comes from the accumulation step. Generally, *r*×*l *is much larger than *g *to increase coverage. Therefore, Light-RCV is generally much faster than the for-loop approach.

During the read coverage construction, the mismatch information is also extracted. Such information in SAM file looks like "59A15", which means that on the read of 75 bp long, the 60th position is a mismatch. The "A" shows the nucleotide type at the position on the reference genome. Figure 1(l) indicates a mismatch.

### Multi-resolution profiles

Since the chromosome size can be up to 100 million base pairs, visualizing a whole chromosome is slow and may crash NGS viewers if the memory arrangement is not carefully designed. To solve this problem, Light-RCV generates multiple profiles (i.e. read coverage curves) with different scales for each chromosome. The first profile is at the base level, in which a data point represents a bp in the genome. This profile is used when the user selects a viewing range smaller than 20000 bps. The size of the second profile is 1/20 that of the first one. A data point of the second profile represents 20 bps in the genome, the values (such as read count of the positive strand) are the maximum value of the corresponding 20 bps. This tract is used when the user selects a viewing range of 20001~400000 bps. The third profile is 1/20 of the second one, so on and so forth. As a result, the number of total profiles is dynamically determined by the chromosome size. This design ensures that Light-RCV shows at least 20000 data points at a time, which is feasible for most screens, while minimizing the required resource and the processing time. Moreover, the storage requirement does not greatly increase. The requirement is only approximately 1.05 (= 1 + 1/20 + 1/400 + ...) times of the original required storage.

### Two-stage architecture

**Figure 4:**
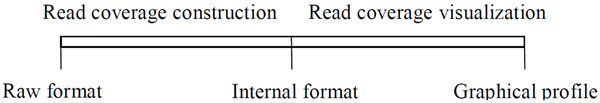
**Two-stage architecture of Light-RCV**. Two-stage design of Light-RCV for NGS data processing, composed of read coverage construction stage and read coverage visualization stage.

In Light-RCV, most computations in the flow of read alignments to a read coverage are moved to a separate stage that is relatively less critical to UX. Namely, the process is split into two stages (Figure 4). Existing NGS viewers do not explicitly separate the two stages, where the entire two stages are conducted after users specify a genomic range. This leads to a considerable waste because read coverage construction is required only once for an NGS experiment but users usually specify a genomic range many times. The first stage, which is denoted the "compilation stage" in Light-RCV, prepares the base-level read coverage of the entire genome to an internal format. The second stage, which is denoted the "visualization stage" in Light-RCV, retrieves and visualizes the desired part when users specify a genomic region. In Light-RCV, the internal format is stored in files with an efficient format described in the next subsection. All following visualization operations start from the internal files, even after the computer reboots. The two stages in Figure 4 do not correspond to the backend and frontend described above. Both stages depend on the backend to access the data (mainly writing in the compilation stage and reading in the visualization stage) and on the frontend to interact with users.

### Storage format

To optimize the response time, most computations should be moved to the compilation stage. This computation arrangement is determined by the design of the internal format in Figure 4. The internal file of Light-RCV was designed as the exact format of the base-level read coverage in memory, which is the so-called "memory dump." This design has two important features. First, the data can be retrieved from a specific genomic position without sequentially loading the data before that position. Second, a continuous range of data can be retrieved in one operation without depending on the range size.

## Competing interests

The authors declare that they have no competing interests.

## Authors' contributions

TL and DTHC conceived the research topic, developed the algorithm and wrote the manuscript. JT provided essential guidance. CWC and WBL implemented the program. ACD helped to test the program. All authors read and approved the final manuscript.

## Declaration

Publication charges for this article were funded by Ministry of Science and Technology, Taiwan (NSC 102-2221-E-006-085-MY2).

This article has been published as part of *BMC Bioinformatics *Volume 16 Supplement 18, 2015: Joint 26th Genome Informatics Workshop and 14th International Conference on Bioinformatics: Bioinformatics. The full contents of the supplement are available online at http://www.biomedcentral.com/bmcbioinformatics/supplements/16/S18.
